# Terminological discrepancies and novelties in the histological description of the female genital system: proposed amendments for clinical-translational anatomy

**DOI:** 10.1007/s12565-024-00772-8

**Published:** 2024-04-29

**Authors:** Ivan Varga, Niels Hammer, Lada Pavlíková, Amelie Poilliot, Martin Klein, Renáta Mikušová

**Affiliations:** 1 Faculty of Medicine, Institute of Histology and Embryology, Comenius University in Bratislava, Spitalska Street 24, 842 15 Bratislava, Slovak Republic; 2 Division of Macroscopic and Clinical Anatomy, Gottfried Schatz Research Center for Cell Signaling, Metabolism and Aging, Medical University of Graz, Graz, Austria; 3 Department of Orthopedic and Trauma Surgery, University of Leipzig, Leipzig, Germany; 4 Medical Branch, Fraunhofer Institute for Machine Tools and Forming Technology (IWU), Chemnitz, Germany; 5 Faculty of Health Care Studies, University of Western Bohemia, Pilsen, Czech Republic; 6 Institute of Anatomy, University of Basel, Basel, Switzerland

**Keywords:** Eponyms, Female genital system, Histological controversies, Ovarian follicles, Telocytes, Terminologia Histologica, Tubal lymphatic lacunae, Uterine NK cells

## Abstract

Histological terminology of the female genital organs is currently a part of the internationally accepted nomenclature *Terminologia Histologica* (TH)*,* the latest edition of which dates back to 2008. Many new discoveries have been documented within 16 years since then, and many discrepancies have been found. This paper aims to revise the terminology from clinical and educational perspectives comprehensively. The authors thoroughly searched the current edition of “Terminologia Histologica: International Terms for Human Cytology and Histology,” focusing on missing and controversial terms in the chapter Female genital system. The authors identified six controversial and ambiguous terms and four missing important histological terms. The authors also discussed the addition of less used eponymic terms in the histological description of female genital organs like Hamperl cells, Popescu cells, Kroemer lacunae, Balbiani bodies, Call–Exner bodies, membrane of Slavianski, nabothian cysts, or anogenital sweat glands of van der Putte. We expect the second and revised edition of the TH to be published soon and hope that the Federative International Program on Anatomical Terminology will approve and incorporate all these propositions and suggestions. We also strongly recommend using the official internationally accepted Latin and English histological nomenclature—the TH, either in oral or written form, both in theoretical and clinical medicine.

## Introduction

Terminology is an essential tool for science communication and knowledge transfer. Each field in medicine uses its own terminology, which should also be “comprehensible” for experts from other fields in medicine and allied disciplines. Moreover, misunderstandings can occur when the terminologies of different fields of medicine refer to identical structures of the human body, diseases or therapies using fundamentally different terms. Individual terminologies of distinct areas of medicine should be complementary, and Latin and Greek languages play a pivotal role in these terminologies. Latin terms are fundamental in communication in medicine across multiple languages as most practising doctors in medicine understand Latin terminology or names of diseases using the Latin nomenclature. Unfortunately, nowadays teaching Latin and Greek medical terminology is not adequate in the medical curriculums in numerous countries (Lindekleiv 2005). The future will therefore show what fate awaits the Latin and Greek languages in relation to medical terminology.

The effort to create a uniform and internationally accepted anatomical terminology has a long-lasting history of almost 130 years dating back to the *Basiliensia Nomina Anatomica* established in 1895. Consequently, a number of international committees had been working on preparing a unified final version of anatomical terminology. Such effort was of great importance for the formation of national anatomical terminologies around the world (von Dorsche 1981; Drukker and Walvoort 2000; Gielecki et al. 2008; Kachlik et al. 2008, 2009).

The creation and unification of complex histological terminology was first attempted in 1923. That year, the imperative to create a uniform histological terminology was addressed by anatomists at 32nd congress of the Anatomische Gesellschaft (the Society of German-speaking Anatomists). However, the tangible result of this effort is not generally known to the public (Varga et al. 2018a).

The seventh congress of the International Federation of Association of Anatomists (IFAA) held in New York in 1960 decided to create the *Nomina Histologica*. At the ninth congress of the IFAA in Leningrad (1970), an encompassing list comprising 2846 histological terms was proposed. In the following years, the *Nomina Histologica* was repeatedly revised and new terms were added, including terms from veterinary histology (Tavares de Sousa 1978). With the creation of the Federative Committee on Anatomical Terminology (FCAT) in 1989, terminological work has been restored. A worldwide unification of the morphological nomenclature appears to be a long-lasting endeavour of the Federative International Program on Anatomical Terminology (FIPAT), a working group of the IFAA (Varga et al. 2019). FIPAT develops, publishes and maintains the set of international standard terminologies of human anatomical sciences, the IFAA Terminologies. The official terminology is in Latin, but FIPAT also publishes English equivalents and encourages the IFAA Member Societies to publish translations of the IFAA terminologies in other languages (FIPAT 2024). The IFAA approved the last revision of the Latin histological nomenclature as the only valid official Latin nomenclature for histology in 2003. Five years later, it was issued under *Terminologia Histologica* (TH), in which 4547 terms were listed (Varga et al. 2018a, b, c). It is important to note that such terminology is not immutable—it is a “living organism in development” that is supplemented and improved owing to the suggestions from scientists from all over the world (e.g. Varga et al. 2020; Neumann and Neumann 2021; Neumann et al. 2024).

*Terminologia Histologica*: International Terms for Human Cytology and Histology (FIPAT 2008) is a terminology on human cells, tissues and microscopic anatomy. The TH present the Latin term for each structure accompanied by the term in current usage in English-speaking countries (Allen 2009). The list of items in this still valid, official nomenclature of tissue structures is the best and most extensive of all the histological nomenclatures ever issued.

This article aims to revise the terms associated with the female genital organs in the internationally accepted nomenclature TH from different points of view (e.g. clinical practice or routine histological education). All these activities are aimed at one goal: simple and unambiguous communication in our increasingly globalized world.

## Materials and methods

A systematic and in-depth analysis of the current internationally accepted nomenclature “Terminologia Histologica: International Terms for Human Cytology and Histology” (FIPAT 2008) was conducted. The current version of TH comprises 189 histological terms associated with the female genital system. The analyses focused on finding missing or controversial terms and ambiguous terms related to tissue structures associated with the female genital system, as well as less often used eponymous terms. The analysis was based on many years of experience as teachers of anatomy and histology at various European universities, as well as on our experience as scientists who study cells and tissues within the female genital system. We also discuss the significance of our findings for everyday practice and communication among morphologists, clinicians and educational purposes.

## Results and discussion

### Controversial or ambiguous histological terms

#### Developmental stages of ovarian follicles

The recent TH describes five developmental stages of ovarian follicles:

- Primordial ovarian follicle—including primary oocyte and simple squamous epithelium.
- Primary ovarian follicle—including primary oocyte, zona pellucida and simple cuboidal epithelium.
- Secondary ovarian follicle—including primary oocyte, zona pellucida, stratified cuboidal epithelium and follicular theca.
- Tertiary (vesicular, antral) ovarian follicle—including primary oocyte, zona pellucida, granulosa, follicular antrum with follicular fluid and theca interna and theca externa.
- Mature (preovulatory) follicle—including secondary oocyte inside a corona radiata and cumulus oophorus.

Upon review of the standard literature on histology, anatomy and reproductive medicine textbooks, one will find that except for the term “primordial follicle”, the other developmental stages of ovarian follicles are described (usually) in entirely different ways (Table 1). Interestingly, only 1 single textbook (Mills 2020) of the 16 analysed adheres to the recommended terminology according to FIPAT (2008). Moreover, in one textbook (Standring 2021), different developmental stages are described in the main text, but completely different ones are shown and described in the schematic drawings within the same chapter. Such discrepancies underline the necessity of finding a compromise and proposing a terminology that best describes the developmental stages of ovarian follicles, respecting current knowledge and traditions in nomenclature.

**Table 1:** Description of ovarian follicles’ developmental stages according to different histological, embryological and anatomical textbooks

Source/textbook	Stages of ovarian follicle development
*Terminologia Histologica* (FIPAT 2008)	Primordial—primary—secondary—tertiary (vesicular, antral)—mature (preovulatory) follicle
Eroschenko. diFiore’s Atlas of Histology (2013)	Primordial—primary (with simple or stratified epithelium)—secondary (antral)—mature follicle
Ovalle and Nahirney. Netter’s Essential Histology (2013)	Primordial—primary—preantral secondary (several layers of granulosa cells)—late term secondary (vesicular, antral)—tertiary (mature, Graafian) follicle
Young et al. Wheater’s Functional Histology (2014)	Primordial—early primary (single layer of cuboidal follicular cells)—primary (several cell thick layers of granulosa cells)—secondary (with cavities)—Graafian follicle (with secondary oocyte)
Kierszenbaum and Tres. Histology and Cell Biology (2016)	Primordial—primary (unilayered)—secondary (multilayered)—preantral—antral—preovulatory (Graafian) follicle
Mescher. Junqueira’s Basic Histology (2016)	Primordial—unilaminar primary—multilaminar primary—secondary (antral)—mature (Graafian) follicle
Gartner. Textbook of Histology (2017)	Primordial—unilaminar primary—multilaminar primary—secondary (with spaces between granulosa cells)—Graafian follicle
Sadler. Langman’s Medical Embryology (2019)	Primordial—early primary—mature primary (preantral, with stratified granulosa cells)—vesicular (antral)—mature vesicular (Graafian) follicle
Lowe et al. Stevens & Lowe’s Human Histology (2020)	Primordial—unilaminar primary—multilaminar primary—secondary (with fluid-filled cavity)—tertiary (Graafian) follicle
Mills. Histology for Pathologists (2020)	Primordial—primary—secondary (preantral)—tertiary (antral, vesicular)—mature (Graafian) follicle
Moore et al. The Developing Human (2020)	Primordial—primary—secondary (vesicular)—tertiary (mature) follicle
Girsh. A textbook of Clinical Embryology (2021)	Primordial—primary—secondary—preantral—early antral—mid antral—preovulatory (Graafian) follicle
Schoenwolf et al. Larsen’s Human Embryology (2021)	Primordial—primary—growing—antral (vesicular)—mature vesicular (Graafian) follicle
Standring. Gray’s Anatomy (2021)	*Description in text:* primordial—primary—secondary—antral—preovulatory (tertiary, Graafian) follicle *Description of a schematic drawing:* primordial—early primary—late primary—secondary (antral)—tertiary (Graafian) follicle
Paulsen. Histology and Cell Biology (2022)	Primordial—unilaminar primary—multilaminar primary—secondary (with fluid-filled cavities)—mature (Graafian) follicle
Carlson. Human Embryology and Developmental Biology (2024)	Primordial—primary—multilayered preantral—secondary antral—large antral—tertiary (Graafian) follicle
Pawlina. Histology (2024)	Primordial—early primary follicle (single layer)—late primary follicle (multilayered mass of granulosa cells)—secondary follicle (with fluid-containing antrum)—mature Graafian follicle

A description of the developmental stages of the follicles is easier in clinical practice when compared to the histological description only. In gynaecological practice, ultrasound is the most widely used approach to perform real-time ovarian imaging in patients (and animals). Vaginal ultrasound is suitable for detecting large antral follicles; however, it is challenging to image preantral follicles in women (Feng et al. 2018). That is why, especially for clinical needs, dividing the follicles into preantral and antral appears sufficient. Another clinical approach is folliculometry (i.e.: the sequential sonographic monitoring of both the number and dimensions of the follicular cohort) which is used to determine ovulation. In this method, to correctly measure follicular size relevant to assisted reproduction techniques is essential since follicles of 10 mm or larger (total follicular number) offer relevant information on the risk of ovarian hyperstimulation syndrome. Follicles larger than 14 mm are used to calculate the number of expected mature oocytes at retrieval; and follicles larger than 18 mm are used to determine the time of ovulation triggering (Rodriguez et al. 2014). In ultrasound, the presence of a cavity in the follicle (preantral and antral stage) and the size of follicles are essential, not the histological stages of development. Additionally, according to same clinicians, the term Graffian follicle is only a follicle protruding/bulging on the surface, which makes it different form the less developed tertiary/mature one.

Keeping in mind that any terminology is useless without worldwide acceptance, it is counterproductive to promote one’s ideas when they do not find their way into textbooks for 15 years—as evidenced by the analysis of 16 internationally recognized textbooks observed in Table 1. Based on a compromise from world textbooks, the authors recommend using the following designations of developmental stages of ovarian follicles:

- primordial ovarian follicle,
- unilaminar primary ovarian follicle,
- multilaminar primary ovarian follicle (preantral),
- secondary ovarian follicle (antral),
- mature (preovulatory, tertiary) ovarian follicle (eponym—Graafian follicle).

#### Haemorrhagic body of the ovary

During the normal ovarian cycle, the antrum of mature follicle after ovulation fills with blood originating from the ruptured capillary bed within the *theca interna*. According to TH, the official term for this initial stage of *corpus luteum* (yellow body) development is a haemorrhagic body (*corpus haemorrhagicum)* or *corpus rubrum*. The blood soon forms a clot, which is usually mild and limited in extent. Under normal conditions, this state is clinically irrelevant and resolves quickly. If fertilization does not occur, the *corpus luteum* will degenerate and the cycle repeats. However, if the bleeding becomes excessive, the *corpus luteum* enlarges into a cystic structure, which may rupture, causing acute bleeding into the abdominal cavity. This condition is called haemorrhagic *corpus luteum* (Medvediev et al. 2020). Using the term “haemorrhagic” is commonplace in other papers as well. For instance, Gupta et al. (2015) also reported “*corpus luteum* haemorrhage” in patients on anticoagulation therapy. The same terminology was used in a case report by Jarvis and Olsen (2002), who described cases of “recurrent *corpus haemorrhagicum*” in women suffering from von Willebrand’s disease. Hatipoglu et al. (2014) identified “*corpus haemorrhagicum* cyst rupture” as a possible cause of acute right lower abdominal pain in female patients of reproductive age. The term *“corpus haemorrhagicum*” from the sense of gynaecologists and surgeons in women of reproductive age forms a gynaecologic pathology, which may be misleading in the diagnosis of acute appendicitis. Other surgical journals also describe cases of acute abdomen due to a *corpus haemorrhagicum* (e.g. Colak et al. 2001; Kadikoylu et al. 2010). These studies from different periods over the last 25 years demonstrated that the term “*corpus haemorrhagicum*” has a specific pathological meaning in clinical practice. One can see that there is a significant terminological overlap between a term for benign physiological bleeding into the cavity of the forming *corpus luteum* and a potentially life-threatening condition which may lead to an abdomen with acute haemoperitoneum. “Haemorrhage” by itself is a term which is almost always associated with a pathological condition, rarely ever describing a normal physiological state of any bodily organ/structure. Therefore, the authors propose to change this term in the revised edition of the TH. *Corpus rubrum* should be the preferred term, since it does not have this pathological connotation. The authors recommend using the following designations of developmental stages of follicle during and after ovulation: *folliculus ovaricus matures—corpus rubrum—corpus luteum—corpus luteolyticum/luteum degenerans—corpus albicans*.

#### Fusocellular connective tissue of ovaries

Fusocellular connective tissue (*textus connectivus fusocellularis, textus connectivus spinocellularis*), a component of the ovarian stroma, is also a new and unexplained term in TH. However, this type of connective tissue and the term “fusocellular” is usually mentioned in the scientific (mainly clinically oriented) literature for some types of ligaments and fascia (e.g. Kumka and Bonar 2012; Freiwald et al. 2016) as well as when describing tumours, as leiomyomas or fusocellular sarcomas (Sousa et al. 2009; Goker et al. 2022; Montoya-Beltran et al. 2023). The authors of this new term probably attempted to highlight the high presence of fusiform or spindle-shaped cells (primarily fibroblasts) in the stroma of ovaries. However, similar-shaped cells are generally present in the connective tissue proper, i.e.: the connective tissue layer of the endometrium is extremely “cellular” and is made up mostly of fusiform fibroblasts—a population of precursor cells, which further differentiate into decidual cells after implantation of the embryo (Varga et al. 2019). It is therefore unnecessary to introduce a new type of connective tissue proper—especially if its definition is imprecise and is misleading for pathologists. The authors recommend deleting the term fusocellular connective tissue from TH.

#### The spongy layer of the vagina

It is well known that the deeper areas of the vaginal mucosa contain numerous thin-walled veins. During sexual arousal, vaginal blood flow increases, resulting in vasocongestion. This in turn raises the vaginal surface temperature and enhances vaginal transudation and lubrication, thus, facilitating painless penile penetration and creating erectile tissue tumescence, which sensitizes the vagina (and similarly clitoris and labia) to stimulation leading to orgasm (Levin 2015). In recent histology textbooks, this specialized connective tissue layer of the vagina filled with blood vessels is considered as being a part of the vaginal mucosa (the subepithelial lamina propria). But lamina propria of vaginal wall in the recent TH is missing. Less often, it is considered a separate submucosa (but the exact border between connective tissue mucosa and connective tissue submucosa is unclear due to the absence of the *lamina muscularis mucosae* in the vaginal wall). It can be presumed that the new term “spongy layer of the vagina” *(tunica spongiosa)* in TH originated from its resemblance to the erectile tissue of the clitoris, the *corpus spongiosum clitoridis,* or this concept can be derived from a similar concept of the urethra. The authors would like to add three considerations regarding the new term “spongy layer of vagina”:

- According to Puppo and Puppo (2015), the vagina has no direct morphological relationship with the clitoris. Therefore, there is no need to use similar terminology when describing the vaginal wall and the microscopic structure of the clitoris. On the other hand, other scientists clearly describe “clitourethrovaginal complex” as a distinct anatomical entity (Wei et al. 2023),
- TH lacks a clear differentiation whether this vaginal spongy layer is identical to the lamina propria of other hollow organs or if there is a separate mucosa of the vagina, and the spongy layer lies underneath the mucosa as a distinct second layer of the vaginal wall (Varga et al. 2019). If the latter statement is true, the spongy layer should be identical to a hypothetical “vaginal submucosa” (true submucosa is present only in a digestive tube, where a clear border is detectable between the lamina propria and submucosa, based on the presence of *lamina muscularis mucosae*).
- It is also necessary to keep in mind that the structure of the vaginal wall is not uniform. According to Hoag et al. (2017), there is no apparent erectile or “spongy” tissue in the anterior vaginal wall, except a single spot/area where the urethra abuts the clitoris distally.

Until now, the new term “spongy layer of vagina” was not transferred to histology textbooks and this term was not identified in any of the histology textbooks mentioned in Table 1. Our opinion is that the spongy layer of vagina is a functionally important and distinct structure and as such it has to be named and mentioned in TH; otherwise, the TH would be imprecise and too traditional. Therefore, we think that the description of the vaginal wall should be as follows:

- mucosa (including epithelium and lamina propria),
- spongy layer (rich in thin-walled veins),
- muscle layer,
- adventitia.

#### Basal cells of tubal epithelium: myth or reality?

The description of the uterine tube epithelium also contains a cell population called basal epitheliocyte (*epitheliocytus tubarius basalis*). The presence of basal, undifferentiated cells in the tubal epithelium has long been a cytological enigma and was first described by Pauerstein and Woodruff (1967). These authors assumed these cells to represent reserve cells for tubal epithelial or connective tissue cells. Scientific literature named epithelial cells responsible for tubal epithelial regeneration and in pathological conditions for initiation for high-grade serous ovarian carcinoma as “uterine tube epithelial stem-like cells” (Paik et al. 2012) or “progenitor basal stem cells” (Zhu et al. 2020). Our observations for change in TH are based on three points:

- Under the term “basal cell” of tubal epithelium, most morphologists imagine small polyhedral cells similar to “basal cells” of respiratory epithelium or epididymal duct.
- Within the tubal epithelium, most of small and round cells with dark nuclei and pale cytoplasmic halo localized near the basement membrane are intraepithelial T-lymphocytes (Odor 1974; Peters 1986; Varga et al. 2018b).
- Stem cell for tubal epithelium regeneration are morphologically more similar to gastrointestinal stem cell *(cellula gastrointestinalis precursoria)*.

We propose to replace term “basal epitheliocyte” with “tubal epithelial stem cell” and add a new term, “intraepithelial T-lymphocyte” in TH. These intraepithelial regulatory T-lymphocytes have a huge functional importance in reproduction (suppression of the immune response against both sperm and semi-allogeneic embryos) and classify the uterine tube as an immunoprivileged organ (Visnyaiová et al. 2024).

#### Is the term “endometrial granular cell” suitable?

The first edition of the TH mentions a functionally important population of endometrial granular cells. Unfortunately, this term is seldom used in clinical practice. In clinical practise, these cells are named “uterine NK cells” and are crucial in maintaining physiological gestation. Uterine NK cells yield critical immunomodulatory functions with the potential to control embryo implantation and trophoblast invasion, regulate placental vascular remodelling and promote embryonic/foetal growth (Lapides et al. 2023). On top of that, some authors describe separate endometrial NK and decidual NK cells as those found in the endometrium after decidualization (Xie et al. 2022). Therefore, “uterine NK” instead of “endometrial NK” appears to be a more suitable term as it is an umbrella term for both NK cells in endometrium before and during pregnancy (in decidua). Additionally, the revised TH edition can also include the eponym ‘Hamperl cells’ after Herwig Hamperl (1899–1976), who first described them (Gross et al. 2019).

### Missing important histological terms

#### Cohort of follicles/follicular cohort

The cohort of follicles/follicular cohort (*cohors folliculorum*) is a term missing in the current edition of the TH. From our perspective, this is surprising, given its ubiquitous usage in contemporary scientific literature (especially those focused on reproductive medicine techniques). The cohort of follicles is a technical term for a group of six to twelve primordial ovarian follicles that develop jointly at the beginning of a new ovarian cycle due to elevated follicle-stimulating hormone levels. After searching the various databases, a paper published in 1976 was found using this term (Welschen and Dullaart 1976), as well as numerous articles from recent years studying the ovaries via ultrasound as a part of in vitro fertilization (e.g. Steiner et al. 2023; Friis Wang et al. 2023; Maghraby et al. 2024). This term is also widely used in histology and assisted reproductive technique textbooks (e.g. Balko et al. 2018; Gardner et al. 2024). Considering its wide usage in clinical and experimental settings and teaching practice, the term “cohort of follicles or follicular cohort (*cohors folliculorum*)” should be included in the revised edition of the TH. For completeness, usually, only one follicle from the cohort of follicles in the monovulatory species (as humans, cows and horses) completes its development, which is referred to as the “dominant follicle (*folliculus dominans*)” in the clinically oriented scientific literature (Ginther 2021; Casarini et al. 2022). However, this is another missing term in the current histological nomenclature.

#### Uterine, tubal and vaginal telocytes

The TH does not mention telocytes in female reproductive. Telocytes are interstitial cells with small bodies and very long cytoplasmic projections with many different functions. In the uterus and uterine tubes, these cells were first described as “interstitial Cajal-like cells” in 2007 (Popescu et al. 2007). Later, telocytes were also found in the uterine cervix (Klein et al. 2017) and vagina (Rosa et al. 2023). Telocytes are also implicated in coordinating and regulating muscle contractility (Creţoiu et al. 2012; Zhu et al. 2023), but they were also found to perform important immunological tasks related to the maternal immune tolerance of the hemi-allogeneic embryo (Klein et al. 2022) or enhanced the proliferation of endometrial stromal cells and regeneration of endometrium (Tang et al. 2019; Chen et al. 2023). They are essential interstitial regulators responsible for cell-to-cell communication and coordination between other stromal cell populations (Cretoiu and Cretoiu 2016). From a clinical perspective, telocytes may play an essential role in the pathogenesis of tubal ectopic pregnancy (Karasu et al. 2022), uterine fibroids (leiomyomas) (Varga et al. 2018b; Aleksandrovych et al. 2019) or endometriosis (Xu et al. 2023). Therefore, telocytes deserve a place in the new edition of TH. Since the discovery of telocytes is clearly linked to the research team around the Romanian morphologist Laurentiu Mircea Popescu (1944–2015), we also propose the eponymous name of telocytes as “Popescu cells”.

#### Central lymphatic lacunae of tubal fimbriae and mucosal folds

**Fig. 1:**
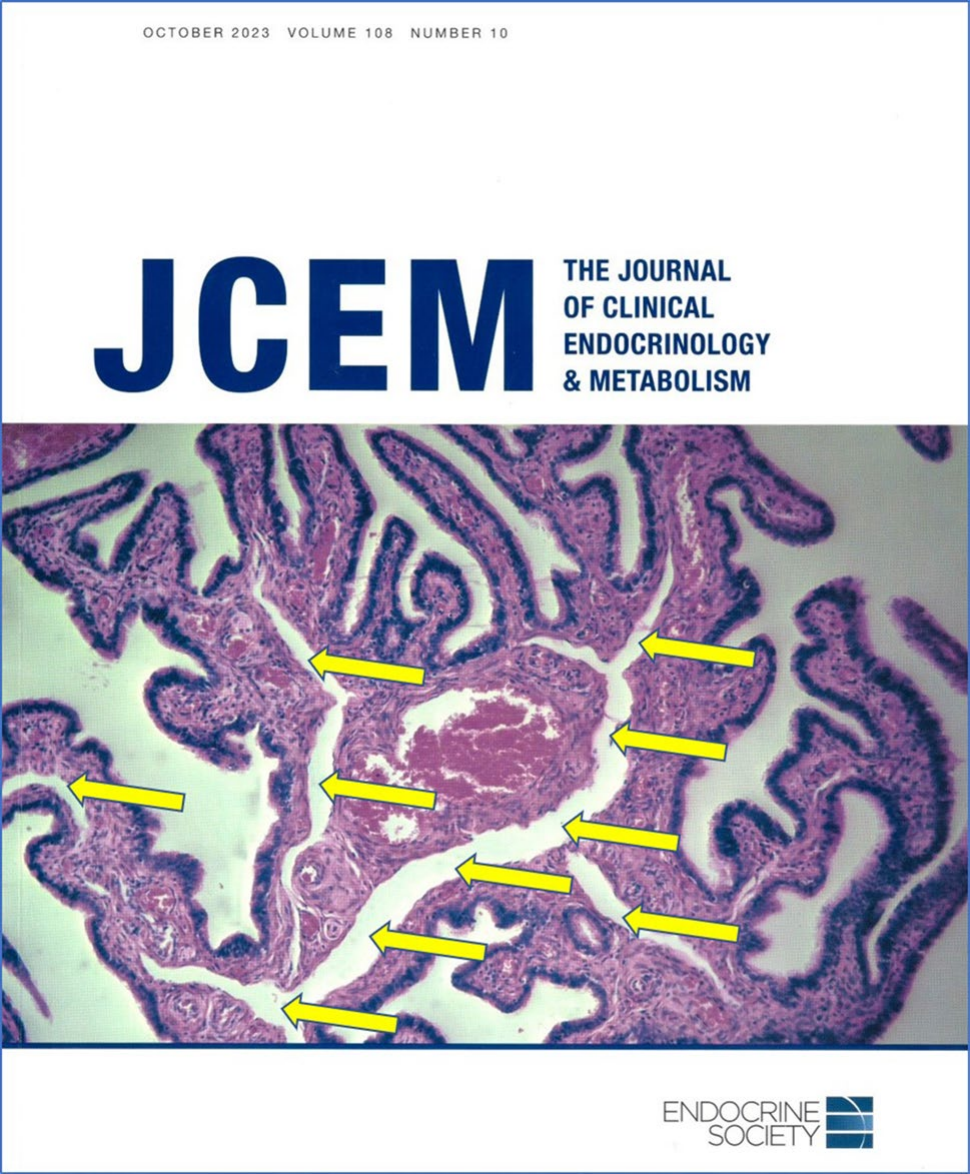
The cover page of the Journal of Clinical Endocrinology & Metabolism (October 2023) with a histological image of human uterine tube mucosal folds. We highlighted well-visible central lymphatic lacunae with yellow arrows

Another missing term is related to the specifics of the lymphatic drainage of the uterine tubes. For more than 120 years, histologists have overlooked a peculiar feature of the tubal mucosal folds. The sketches in old histological textbooks and photomicrographs of tissue sections stained with regular methods demonstrate that each uterine tube mucosal fold contains wide spaces. These were first described in a habilitation thesis by a German gynaecologist Paul Kroemer in 1904, who thought of these spaces as lymphatics, naming them “Lymphbahnen” in German, meaning lymphatic channels (Kroemer 1904). Strikingly, they are usually overlooked in textbooks or mentioned only marginally without discussing their significance or function despite their evident visibility, even in the most basic of histological stains (Fig. 1). Our previous research demonstrated immunohistochemically that these wide spaces are really lymphatic, as Kroemer first suggested. Consequently, they were termed central lymphatic lacunae of tubal mucosal folds following their precise histological description via a palette of different immunohistochemical markers for lymphatic endothelial cells such as l podoplanin (D2-40) and VEGFR-3, and in scanning electron microscopy (Varga et al. 2018a, b, c; Csöbönyeiová et al. 2022). The authors also hypothesized about their probable functions, including tubal fluid maintenance, vital for normal reproductive ability. The lymphatic lacunae in the fimbriae also probably work like an erectile tissue, enlarging the fimbriae and making it easier for the uterine tube to capture an oocyte during ovulation. Considering the abovementioned elements, lymphatic lacunae should be included in the official histological nomenclature. Based on the first description of these tubal mucosal histological structures, we suggest an eponym: “Kroemer lymphatic lacunae” of the uterine tubes.

#### Squamocolumnar junction of the uterine cervix

The squamocollumnar junction of the uterine cervix appears to be another missing term of high clinical significance. As the name suggests, this junction demarcates the transition found in the epithelial lining of the endocervical and exocervical mucosa, which is simple columnar and stratified squamous non-keratinized, respectively. This site of abrupt change in the type of epithelial lining is of great concern in gynaeco-oncology. The cellular origin of most cervical cancers is thought to be cells at the squamocolumnar junction of the uterine cervix. Approximately, 95% of cervical cancers are caused by human papillomavirus (HPV) infection. Cervical cancer by itself is the fourth most common cause of cancer in women (Kusakabe et al. 2023; Jain and Limaiem 2024). This junction between two types of lining epithelia is also visible during colposcopic gynaecological examination (Mergui et al. 2023). What is typical of this junction is its dynamic nature. Its location is not static, but changes according to age, parity, hormonal balance and other variables (Singer 1975; Carcopino et al. 2008).

A distinct term closely related to the squamocolumnar junction is the transformation zone. This term is sometimes erroneously used as a synonym for the junction, but in fact it describes a zone of metaplastic epithelium, which results from the transformation of the ectopic endocervical epithelium into the stratified squamous non-keratinized epithelium. Its clinical significance lies in the fact that the forming transformation zone is prone to HPV-induced changes (Prendiville and Sankaranarayanan 2017). Considering the revised edition of the TH, we propose both terms to be added because of their importance in clinical practice.

## Less used eponyms in histological description of female genital organs

The eponyms in anatomical and medical terminologies have as many supporters as they have detractors. An eponym is a type of synonym for an anatomical structure that is named after a person. Hundreds of structures have an eponym in addition to the official descriptive term. Physicians and surgeons are used to using eponyms in descriptions of diseases and clinical procedures, but anatomical eponyms are frowned upon by anatomists because they convey no descriptive information about a structure’s location in the body or its other identifying characteristics (Gobée et al. 2024). According to many anatomists, introducing any eponym is against the rules of anatomical nomenclature which has excluded all eponyms already in 1955. Although the eponyms are officially contraindicated in anatomical terminology, they are still placed in anatomical and other medical books (Burdan et al. 2016).

Some example of eponyms associated with the description of gross anatomical structures of female reproductive system are Fallopian tube (uterine tube), Bartholin gland (greater vestibular gland), Skene glands (lesser vestibular/paraurethral glands located on either side of the opening of female urethra), Halban fascia (layer of dense connective tissue present between the trigone of the urinary bladder and the anterior part of the vaginal wall), Hart line (the junction zone between the labia minora and the vulvar vestibule) or plexus of Kobelt (venous plexus situated until the angle of the clitoris). These eponyms are properly described in gross anatomical articles and textbooks. Similar generally used term in microscopic anatomy is Graafian follicle (mature ovarian follicle). Other eponyms are associated with some anatomical variations or anomalies of the female genital system, e.g. Gartner duct cyst (benign vaginal cyst) or Morgagni hydatid cyst (vesicular appendix of the uterine tube). These eponyms are so widely used in gynaecological practice that warrant no further explanation. Other relatively less known and newly proposed eponyms we mentioned before, such as Hamperl cells, Popescu cells and Kroemer lacunae of uterine tubes. In the following section, we describe several lesser-known eponyms.

The most characteristic ultrastructural morphological feature of dormant oocytes inside primordial follicles of many species is the Balbiani body. Balbiani body is a perinuclear dense collection of mitochondria and other organelles, adjacent to the oocyte nucleus. Sometimes, it is termed “mitochondrial cloud” or a non-membrane-bound super-organelle consisting mostly of mitochondria but also Golgi, endoplasmic reticulum, other vesicles and RNA (Dhandapani et al. 2022). The Balbiani body disassembles during oocyte maturation and may be one of the first requirements for oocyte maturation (Amin et al. 2023). These structures in oocytes were firstly studied intensively by French embryologist Edouard-Gérard Balbiani (1823–1899) in a variety of animal species. The eponymous name “Balbiani vitelline (yolk) body” or recently used “Balbiani body” was given to the structure by Balbiani’s students (Wessel 2012). This structure has no suitable equivalent within the current version of TH and further discussion will be required to determine the official Latin and English terms.

**Call–Exner bodies** are present in ovarian follicles of a range of species including humans, and in a range of human ovarian tumours. A Call–Exner body is composed of a ring (rosette) of granulosa cells disposed radially around a central cavity filled with eosinophilic extracellular matrix/fluid. Electron microscopy has demonstrated that some Call–Exner bodies contain large aggregates of convoluted basal lamina (van Wezel et al. 1999) and the fluid is in electron microscopic level and histochemically (stained positively with Alcian blue or Periodic Acid Schiff reaction) similar to follicular fluid (Rodgers et al. 2000). The role of Call–Exner bodies is unknown. This eponym relates to Sigmund Exner (1846–1926) and Emma Louise Call (1847–1937). Exner is remembered for his important contributions to brain research. Well known is another neuroanatomical eponym, the “Exner area”, a discrete area of the middle frontal gyrus, which was dedicated to the function of writing (Roux et al. 2010). Emma Louise Call was one of the first female doctors in the USA. After receiving her medical degree from the University of Michigan in 1873, she went to Vienna as Exner’s postgraduate student. Her description of Call–Exner bodies was her only publication (Al Aboud and Al Aboud 2014). This structure has no suitable equivalent within the current version of TH and further discussion will be required to determine the official Latin and English terms.

The basement membrane between granulosa cells and thecal cells in the ovarian follicles *(membrana basalis folliculi)* is termed as the “**membrane of Slavianski**”. Kronid von Slavianski/Slaviansky (1847–1898) was the first to find the networks of reticular fibres among theca interna cells in women in 1870 (Yamashita 1960). But this eponym is usually used only in French (“la membrane de Slavianski”), Spanish (“membrana de Slavianski”), Italian (“membrana di Slavianski”) and Slavic languages (“Slavianského membrána”), and we could not find this eponymous term in English literature.

Mucous containing dilated cervical gland of the cervix *(glandula cervicalis dilatata)* is seldom referred to by any other name than n**abothian cyst**, according to the German anatomist Martin Naboth (1675–1721) (Speert 1956). Presence of small-sized nabothian cysts (also called retention cysts) is a common and benign gynaecological condition in reproductive age which has no clinical significance (Nassif et al. 2017).

In 1991, the Dutch pathologist, Sebastian C. van der Putte, described a new variant of cutaneous apocrine glands that occurs in the anogenital region, with its highest concentration in the interlabial sulcus of the vulva (**anogenital glands of van der Putte;** *glandula sudorifera anovulvaris*). Their strict localization around the derivatives of the embryonic cloacal entrance suggests a possible role in sexual functions. This gland, which is characterized by a long excretory duct opening at the skin surface, was characterized by the author as “mammary-like anogenital glands” (van der Putte 1994).

An important historical figure in world of anatomy is undoubtedly the German anatomist Heinrich Wilhelm Gottfried Waldeyer-Hartz (1836–1921). His prolific career is reflected via many anatomical and histological structures being named after him. Arguably, the most known is the pharyngeal lymphatic ring of Waldeyer (Winkelmann 2007). His scientific interest was also focused on the female genital system, as evidenced by an eponym **germinal epithelium of Waldeyer**. This misnomer is based on an outdated notion that female germ cells of the ovary originate in this epithelium. In clinical practice, the most common term is the ovarian surface epithelium, while TH recognizes two equivalent terms: the ovarian mesothelium and surface epithelium. Interestingly, Nishida and Nishida (2006) published a “debate” in which they argued that oocytes can originate from the cells of the surface epithelium so that the term ‘germinal epithelium’ can be reintroduced into terminology. This “discovery” is dubious, but a further discussion of this topic is out of the scope of this paper. Additionally, the surface epithelium of ovaries is also referred to as “**Müllerian epithelium**”—even though there is no embryological link between the ovarian surface epithelium and the Müllerian ducts, from which internal female genital organs other than the ovaries originate (Austria and Dubeau 2023). Both eponyms are named after Johannes Peter Müller (1801–1858), a German anatomist, who described embryonic paramesonephric (Müllerian) ducts.

## Conclusion

In the last revision of the Latin histological nomenclature in the TH, 4547 terms were listed. Such terminology is variable and under constant change—it is a “living organism in development and advancement” that is supplemented and improved thanks to suggestions from scientists from all over the world (e.g. Varga et al. 2020; Neumann and Neumann 2021; Neumann et al. 2024). The second revised edition of the TH is expected to be published soon. One direction of future discussions will be the anglicizing of Latin terms, such as corona radiata to radiate corone; zona pellucida to pellucid zone; cumulus oophorus to oophoric collicle/cumulus; theca interna and externa to internal and external follicular theca, etc. The authors of this article strongly recommend using the official internationally accepted Latin and English histological nomenclature—the TH, either in oral or written form, both in theoretical and clinical medicine.
